# Application and accuracy of the EAPC/IASP diagnostic algorithm for neuropathic cancer pain and quantitative sensory testing profile in patients with pain due to cancer

**DOI:** 10.1097/PR9.0000000000001140

**Published:** 2024-02-16

**Authors:** Morena Shkodra, Matthew Mulvey, Marie Fallon, Cinzia Brunelli, Ernesto Zecca, Paola Bracchi, Mariangela Caputo, Giacomo Massa, Silvia Lo Dico, Roman Rolke, Stein Kaasa, Augusto Caraceni

**Affiliations:** a Palliative Care, Pain Therapy and Rehabilitation Unit, Fondazione IRCCS Istituto Nazionale Tumori, Milan, Italy; b Institute of Clinical Medicine, University of Oslo, Oslo, Norway; c Leeds Institute of Health Sciences, University of Leeds, Leeds, United Kingdom; d Department of Palliative Medicine, University of Edinburgh, Edinburgh, United Kingdom; e Department of Palliative Medicine, Medical Faculty RWTH Aachen University, Aachen, Germany; f Department of Oncology, Oslo University Hospital, Oslo, Norway; g Department of Clinical Sciences and Community Health, Università degli Studi di Milano, Milan, Italy

**Keywords:** Cancer pain, Neuropathic cancer pain, Quantitative sensory testing, Diagnostic algorithm, Somatosensory profiling, NeuPSIG criteria

## Abstract

**Introduction:**

Better diagnosis and treatment of neuropathic cancer pain (NcP) remains an unmet clinical need. The EAPC/IASP algorithm was specifically designed for NcP diagnosis; yet, to date, there is no information on its application and accuracy.

**Objectives:**

Our aim was to determine the accuracy of the EAPC/IASP algorithm compared with the Neuropathic Special Interest Group grading system (gold standard) and to describe patients' sensory profile with quantitative sensory testing (QST).

**Methods:**

This is a cross-sectional observational study conducted in a palliative care and pain outpatient clinic. Patients with cancer pain intensity ≥3 (numerical rating scale 0–10) were eligible. The palliative care physician applied the EAPC/IASP algorithm as a grading system to diagnose probable or definite NcP, and an independent investigator applied the gold standard and performed the QST. Sensitivity and specificity of the EAPC/IASP algorithm were measured in comparison with the gold standard results. Kruskal–Wallis and unequal variance independent-samples *t* tests were used to compare the QST parameters in patients with and without NcP.

**Results:**

Ninety-eight patients were enrolled from August 2020 to March 2023. Sensitivity and specificity for the EAPC/IASP algorithm were 85% (95% CI 70.2–94.3) and 98.3% (95% CI 90.8–100), respectively. Patients with NcP in contrast to patients without NcP showed cold hypoesthesia (*P* = 0.0032), warm hypoesthesia (*P* = 0.0018), pressure hyperalgesia (*P* = 0.02), and the presence of allodynia (*P* = 0.0001).

**Conclusion:**

The results indicate a good performance of the EAPC/IASP algorithm in diagnosing NcP and the QST discriminated well between patients with and without NcP.

## Abstract 2

Supplemental Digital Content is Available in the Text.

An algorithm for neuropathic cancer pain diagnosis was validated in patients with cancer pain, and using quantitative sensory testing, their sensory profiling has been described.

## 1. Introduction

About 55% of patients with advanced cancer experience pain.^34^ Because of the diversity of tissues involved and the contribution of undergoing anticancer treatments, the clinical presentation of cancer pain can be very variable, with rather complex and not yet well understood pathophysiology.^20,27,33^ The contribution of a neuropathic component to cancer pain, arising because of a lesion of the somatosensory nervous system,^18^ should be distinguished from antineoplastic treatment pain.^3^ The pathophysiology of pain directly because of cancer in these patients can be complex, frequently involving a combination of different mechanisms such as inflammatory, neuropathic, and others that develop as the tumor progresses.^19,26,38^ With these limitations, it has been estimated that neuropathic cancer pain (NcP) prevalence varies from a conservative estimate of 20% to a liberal estimate of 40%.^3^ Because cancer pain treatment guidelines^8,13^ recommend specific adjuvant treatments for NcP, the lack of a standardized assessment method for its diagnosis is certainly clinically relevant.^11,25,32^

The criteria used for the diagnosis of neuropathic pain proposed by the Neuropathic Special Interest Group (NeuPSIG) of the International Association for the Study of Pain (IASP) follow the clinical method of history taking, examination, and confirmatory tests.^14^ These approaches have not been widely used in assessing cancer pain, especially because of limitations such as uncertainties on the relative significance of confirmatory tests, especially sensory signs assessment.^12^ A Delphi expert survey study proposed a modified European Association for palliative care (EAPC)/IASP algorithm^7^ on the application of the NeuPSIG criteria for cancer pain assessment. To the best of our knowledge, no experience on the use of the EAPC/IASP algorithm has been published to date.

Quantitative sensory testing (QST) is a psychophysical method that uses controlled stimuli to quantify the somatosensory function and presence of sensory positive or negative signs.^1^ Although not a stand-alone diagnostic test, its utility has been confirmed for the assessment and monitoring of sensory neuropathies and somatosensory deficits, particularly in diabetic and small-fiber neuropathies.^1,2,28,37^ Yet, few studies have shed light on the function of the somatosensory system in patients with cancer, and little is known on QST discriminative role in NcP.^23,24,31^

The aim of this study was to estimate the accuracy of the EAPC/IASP algorithm in assessing NcP, comparing it with a gold standard based upon the application of the NeuPSIG grading system. To better analyze the somatosensory profile of patients and assess the presence of positive or negative sensory signs, the objective examination was complemented by the use of QST.^1^

## 2. Methods

### 2.1. Study design and population

A cross-sectional observational study was conducted from August 2020 to March 2023 at the Palliative Care and Pain Outpatient Clinic of a comprehensive cancer center. In predefined days, consecutive patients attending the clinic were screened for eligibility and were included in the study, if they had a cancer diagnosis and had cancer pain (directly because of tissue damage caused by the tumor or its metastases) according to a pain syndrome checklist classification, which allows to classify pain diagnosis into different pain syndromes associated with tumor lesions or antineoplastic treatment^9^ with a self-reported average pain intensity score ≥3 on a 0 to 10 numerical rating scale (NRS; 0 = no pain and 10 = worst pain imaginable) in both the previous 7 days and 24 hours. Patients had to be able to provide informed consent and be older than 18 years. They were excluded if they had confirmed or suspected pain associated with cancer treatment (ie, chemotherapy-induced peripheral neuropathy [CIPN] or postsurgical pain), unable to poor clinical conditions, were unable to complete the questionnaires required and/or to sit through 60-minute assessment procedure with regular breaks or to understand the information presented in the information sheet had neurological conditions or diseases that could interfere with the examination, and had local skin inflammation or open lesions at the pain site.

### 2.2. Recruitment and informed consent

Eligible patients were given the necessary information describing the study steps using a patient's information leaflet and were asked to sign a consent form. The study protocol and supporting documentation was approved by the INT Research Ethics Committee (INT 200/19) and was conducted in accordance with the Declaration of the World Medical Association.

### 2.3. Assessments

All assessments were performed on the same day. The following data were extracted from patients' records: age, sex, primary cancer diagnosis, presence of locally advanced or metastatic disease and site/s of metastasis, pain site, pain syndrome according to the checklist in use at our palliative and pain clinic,^9,33^ pain flares, and current analgesic and antineoplastic treatments.

### 2.4. Pain evaluation and neuropathic cancer pain diagnosis

#### 2.4.1. Pain etiology

**Figure 1.**
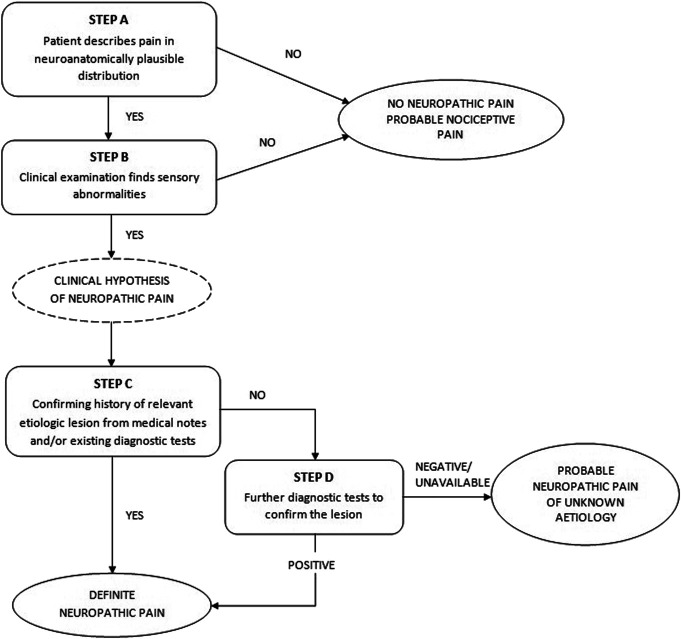
EAPC/IASP algorithm for the diagnosis of neuropathic cancer pain.

**Figure 2.**
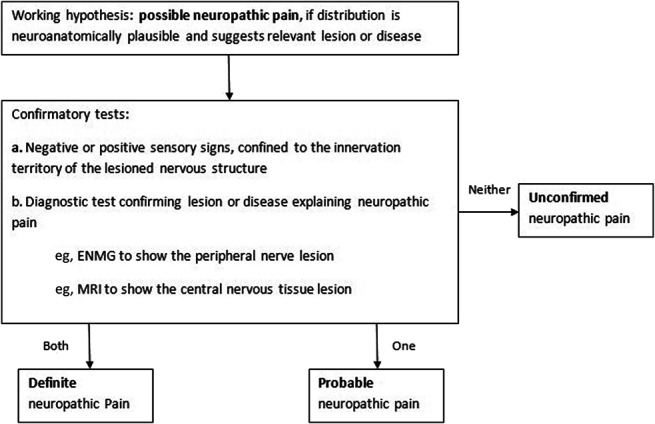
NeuPSIG criteria. ENMG, electroneuromyography; NeuPSIG, Neuropathic Special Interest Group.

Pain etiology was identified by applying the codified pain syndrome checklist,^9^ combining clinical findings with the results of available diagnostic imaging. This classification was used by clinicians to fulfill the algorithm criteria on “relevant etiologic lesion and diagnostic test” (EAPC/IASP algorithm) or “history of relevant lesion or disease” (NeuPSIG criteria) (Figs. 1 and 2).

#### 2.4.2. Definition of test and control sites

Based on the patient's reported information and examination, the site of pain (“test site”) was defined. If the patient had more than one painful area, the region with the most intense pain was selected as test site. A nonpainful control site was identified on the contralateral body region, or if this was painful or the test site was located on the midline, a painless ipsilateral control site above/below the test site was selected also free of neurological findings.

#### 2.4.3. Patient-reported outcome measures

Patients reported pain intensity on the pain site (“test site”) using an NRS from 0 to 10 and completed the self-reported Leeds Assessment of Neuropathic Signs and Symptoms (S-LANSS).^5^

The S-LANSS has 7 questions used to screen for symptoms suggesting the presence of a pain of neuropathic origin. The total score ranges from 0 to 24 points, and scores ≥12 points suggest neuropathic pain.

#### 2.4.4. Neuropathic cancer pain diagnosis

Clinical assessment and NcP diagnosis were performed by 2 independent investigators: the treating physician and a clinical researcher, as described below.

##### 2.4.4.1. Application of the EAPC/IASP algorithm

The EAPC/IASP algorithm^7^ was applied by the palliative care treating physician during the scheduled visit. All palliative care physicians had undergone a brief training session on neuropathic pain assessment and were introduced to the EAPC/IASP algorithm (Fig. 1). Indications and checklists were provided for each of the steps included in the algorithm as described in Appendix A, http://links.lww.com/PR9/A221. Based on the answer provided for each step of the algorithm applied at the pain site, pain was considered neuropathic if there was a clinical hypothesis of probable neuropathic pain.

The clinical examination is described in Appendix A, http://links.lww.com/PR9/A221.

##### 2.4.4.2. Application of the Neuropathic Special Interest Group criteria

After the EAPC/IASP algorithm application, the patient was assessed by another investigator, blind to the previous assessment, who reviewed the electronic medical records, including available diagnostic tests and examined the patient using also a detailed standardized bedside examination for assessment of sensory signs (Fig. 2). It included the following assessments performed in the pain site, comparing with the control site:

(1) Cold and warm sensitivity, assessed using a thermo-roller (Somedic Roll Temp II). For cold, a thermo-roller at 25°C was rolled over a 2-cm length of skin at a rate of 2-second on, 1-second off. For warm, the thermo-roller at 40°C was rolled over a 2-cm length of skin at a rate of 2 second on, 1 second off;
(2) Light touch, assessed using an Owen Mumford Neuropen 10-g monofilament gently pressed against the patient's skin 3 times at a rate of 1 second on, 1 second off;
(3) Pinprick sensation was assessed using an Owen Mumford Neurotip, a disposable needle with a defined pressure of 40 g, pressed against the skin. The Neurotip was placed against the patient's skin 3 times at a rate of 1 second on, 1 second off;
(4) Dynamic mechanical allodynia (DMA), assessed using a cotton bud gently stroked over a 2-cm area of skin. The cotton bud was stroked over the participants pain site 3 times at a rate of one second on, one second off; and
(5) Blunt pressure, assessed using the examiner's thumb pressed against the pain and control sites exerting approximately 4 kg of pressure applied at a rate of 4 kg per second for 4 seconds.

Responses to each test were judged by the investigator, in comparison with the control site, and recorded as “normal”: The participant could feel the stimulus as expected with no flinch or withdrawal of body part; “hypersensitivity”: The sensation was perceived to be more intense than expected; and “hyposensitivity”: The sensation was not felt at all or was less intense than expected.

The bedside examination was considered positive if at least one of the above tests, except for the blunt pressure alone, was abnormal.

Based on the clinical evaluation, available diagnostic tests, and the above bedside examination, the investigator was asked to apply the NeuPSIG criteria.^14^ Pain was considered neuropathic in case of a clinical hypothesis of probable or definite neuropathic pain.

#### 2.4.5. Quantitative sensory testing

Then, the same investigator performed the QST. Each QST session was performed in a quiet room maintained at 22 ± 2°C temperature. Patients were familiarized with the QST procedure by demonstrating the stimuli in a body site that was not the test or control site. A QST battery of tests was adapted based on the standardized protocol of the German Research Network on Neuropathic Pain (DFNS)^29,30^ and was performed at the test (painful) site and then at the control site (Appendix B, http://links.lww.com/PR9/A221). The following parameters were assessed: cold detection threshold (CDT), warm detection threshold (WDT), cold pain threshold (CPT), heat pain threshold (HPT), mechanical detection threshold (MDT), mechanical pain threshold (MPT), pressure pain threshold (PPT), and dynamic mechanical allodynia (DMA).

### 2.5. Statistical analysis

Assuming a prevalence of NcP from 20% to 40% (as per Bennett et al. 2012, *PAIN*^3^), and sensitivity values ranging from 60% to 90%, we calculated that a sample size of 95 patients would allow to estimate a 95% CI half width ranging from 21% to 9%. Frequencies and percentages were used to describe categorical variables, whereas mean values and SDs were used for continuous ones. Point and interval estimates (95% confidence intervals [CIs]) for the prevalence of NcP according to different methods of assessment were calculated. Accuracy of the EAPC/IASP diagnostic algorithm in comparison with the application of the NeuPSIG criteria (gold standard) was assessed by sensitivity and specificity, defined as the chance of a true-positive and true-negative test, respectively,^6^ and estimate precision was measured by 95% CIs. For the QST data, the mean of each parameter was calculated as by the DFNS protocol recommendations.^29^ The within-subject differences between the test and control site for each patient were analyzed. This choice was based on the lack of normative values for some of the body areas analyzed, on the specific characteristics of patients with cancer and on previous evidence of significant sensitivity of such differences.^30^ Within-subject differences were calculated either as a difference of test–control or control–test site, to obtain positive values indicating gain of function and negative values indicating loss of function, depending on the QST variable assessed. To test for the ability of QST to discriminate between patients with and without NcP, unequal variance independent-samples *t* tests were applied to the normally distributed within-subject differences raw data of the QST variables, namely the HPT and CPT. To account for non-normality in the distribution of the rest of the QST variables, nonparametric Kruskal–Wallis tests were applied as sensitivity analysis. *P*-values <0.05 were regarded as significant. This comparison was made only for patients for whom an agreement was found among both evaluations under study. All data analysis was performed using STATA IC 16.^35^

## 3. Results

**Figure 3.**
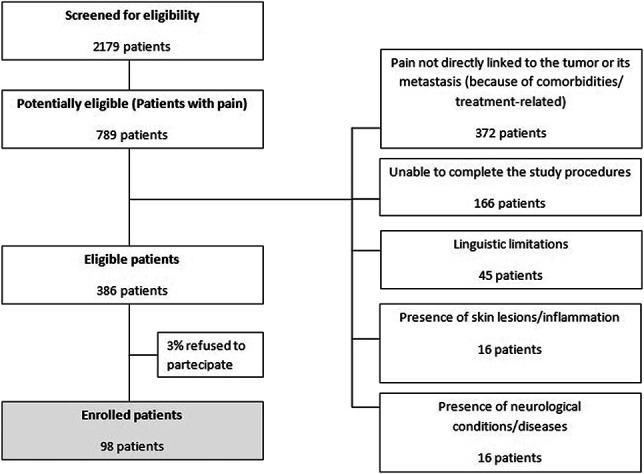
Flowchart of screening and eligibility.

Ninety-eight patients were enrolled and included in the final analysis (Fig. 3). Patients and disease characteristics are reported in Table 1. Mean age was 62.5 years, with 57.1% of patients being female and 42.9% male. The most frequent diagnosis was breast (26.5%) and lung cancer (21.4%). Of the enrolled patients, 47.9% had locally advanced disease and 90.8% metastatic disease. The most frequent metastasis sites were bone (66.3%), lymph nodes (38.8%), liver (25.5%), lung (21.4%), and pleura (9.2%). Regarding antineoplastic treatments, 37.8% were receiving none, whereas 37.8% were receiving chemotherapies, 14.4% hormone therapy, 14.4% immunotherapy, and 16.5% biological and target therapies.

**Table 1:** Patients and disease characteristics (98 patients).

Characteristic	
Age, mean (SD), y	62.5 (13.5)
Sex, N (%)	
Female	56 (57.1)
Male	42 (42.9)
Diagnosis, N (%)	
Breast	26 (26.5)
Lung/bronchial	21 (21.4)
Colon/rectum	7 (7.1)
Prostate	7 (7.1)
Sarcomas	6 (6.1)
Renal	6 (6.1)
Pancreatic	4 (4.0)
Head and neck	2 (2.0)
Uterine	2 (2.0)
Esophagus	1 (1.1)
Thyroid	1 (1.1)
Melanoma	1 (1.1)
Other	14 (14.4)
Locally advanced tumor, N (%)	
Yes	46 (47.9)
No	50 (52.1)
Presence of metastasis, N (%)	
Yes	89 (90.8)
No	9 (9.2)
Metastasis location*, N (%)	
Bone	65 (66.3)
Lymph nodes	38 (38.8)
Liver	25 (25.5)
Lung	21 (21.4)
Pleura	9 (9.2)
Brain	7 (7.1)
Peritoneum	5 (5.1)
Soft tissues	5 (5.1)
Adrenal gland	5 (5.1)
Other	8 (8.2)
Karnofsky performance status mean (SD)	73.6 (5.8)
Antineoplastic treatment*, N (%)	
Chemotherapy	37 (37.8)
Hormone therapy	14 (14.4)
Immunotherapy	14 (14.4)
Other (biological and target therapies)	16 (16.5)
None	37 (37.8)

* More than one is possible.

In Table 2, pain and pain treatment characteristics are described. The mean average pain intensity in the past 24 hours and past 7 days was 6.2 (NRS 0–10) for both measurements, and mean pain duration was 7.1 months. The most frequent pain sites were the back (35.7%) and the pelvis (17.3%), followed by lower limbs (13.3%), abdomen (13.3%), chest (12.2%), and upper limbs (8.2%). Summarizing pain syndromes according to tissue involvement, 68 patients (69.4%) had bone pain, 40 (40.7%) pain because of nervous tissue damage (17 a peripheral nerve syndrome because of soft-tissue or bony tumor, 11 a radiculopathy or cauda equina syndrome because of vertebral lesion, 10 a peripheral nerve syndrome because of chest wall mass, one a brachial plexopathy, and one a cervical plexopathy), 33 (33.7%) pain because of soft-tissue damage, and only 15 (15.3%) patients had visceral pain (Table 2). Of these, 46 patients (46.9%) had just one tissue involvement, and the others had a combination of 2 or more tissue etiologies. Forty patients (40.8%) presented pain flares. The most frequent around-the-clock (ATC) analgesic treatments were fentanyl (33.7%) and oxycodone (30.6%), whereas 7 patients (7.1%) were not receiving any ATC treatments. Adjuvant analgesics included acetaminophen (36.7%), anticonvulsants (35.7%), corticosteroids (32.7%), and NSAIDs (16.3%), whereas 23.5% of the patients were not receiving any adjuvants. Thirty patients (30.6%) had received radiotherapy at the pain site before study enrollment. Mean S-LANSS score for the whole group of enrolled patients was 5.5.

**Table 2:** Pain and pain treatment characteristics (98 patients).

Characteristics	
Average pain intensity in the past 7 d (NRS 0–10), mean (±SD)	6.2 (±1.6)
Average pain intensity in the past 24 h, mean (±SD)	6.2 (±1.7)
Pain duration, mean (SD), mo	7.1 (9.3)
Presence of pain flares, N (%)	
Yes	40 (40.8)
No	58 (59.2)
Pain location, N (%)	
Back	35 (35.7)
Pelvis	17 (17.3)
Lower limbs	13 (13.3)
Abdomen	13 (13.3)
Chest	12 (12.2)
Upper limbs	8 (8.2)
Pain syndrome*, N(%)	
Bone pain	68 (69.4)
Pain because of nervous tissue damage	40 (40.7)
Peripheral nerve syndrome because of soft-tissue or bony tumor	17 (17.3%)
Radiculopathy or cauda equina syndrome because of vertebral lesion	11 (11.2%)
Peripheral nerve syndrome because of chest wall mass	10 (10.2%)
Brachial plexopathy because of tumor invasion	1 (1%)
Cervical plexopathy because of tumor invasion	1 (1%)
Pain because of soft-tissue damage	33 (33.7)
Visceral pain	15 (15.3)
Around-the-clock analgesic treatments*, N(%)	
Fentanyl	33 (33.7)
Oxycodone	30 (30.6)
Codeine	12 (12.2)
Acetaminophen	10 (10.2)
Tapentadol	7 (7.1)
Tramadol	5 (5.1)
NSAIDs	5 (5.1)
Buprenorphine	4 (4.1)
Morphine	1 (1.1)
None	7 (7.1)
Adjuvant analgesics*, N(%)	
Acetaminophen	36 (36.7)
Anticonvulsants	35 (35.7)
Corticosteroids	32 (32.7)
NSAIDs	16 (16.3)
None	23 (23.5)
Radiotherapy at the pain site, N(%)	
Yes	30 (30.6)
No	68 (69.4)
No. of days passed since RT treatment at the time of the visit, mean (±SD)	320 (±78)
S-LANSS score, mean (SD)	5.5 (5.9)

* More than one is possible.

NRS, numerical rating scale; NSAID, nonsteroidal anti-inflammatory drug; RT, radiotherapy.

Prevalence of NcP as estimated by the EAPC/IASP algorithm, NeuPSIG criteria, and S-LANSS was 35.7% (95% CI 26.1–45.4), 40.8% (95% CI 30.9–50.7), and 18.4% (95% CI 10.6–26.2), respectively. Of the 35 patients classified as having NcP by the EAPC/IASP, 34 had a definite and only one a probable diagnosis. However, of the 40 patients classified as having NcP based on the NeuPSIG criteria, 36 had a definite and 4 a probable diagnosis. Sensitivity and specificity of the EAPC/IASP algorithm compared with the gold standard were 85% (95% CI 70.2–94.3) and 98.3% (95% CI 90.8–100), respectively. The lesion considered to cause NcP was a combination of neurological tissue involvement and either bone (51%), visceral tissue (1%), or soft tissue (48%) in the group diagnosed with the EAPC/IASP algorithm and neurological tissue involvement with bone (55%), visceral tissue (0.8%), or soft tissue (44.2%) in the group diagnosed with the gold standard.

### 3.1. Quantitative sensory testing findings

A complete QST profile was available for 93 patients. Five patients had an incomplete profile with at least one of the QST variables available. Quantitative sensory testing examination sensory findings are presented in Table 3. The analysis of the within-subject differences, comparing the painful with the nonpainful site, demonstrated sensory abnormalities in the overall group, with an increased threshold in thermal detection both for cold (CDT) and warm sensation (WDT) (*P* = 0.000) and for heat pain (HPT) (*P* = 0.0369), demonstrating presence of hyposensitivity. Pressure pain threshold (PPT) was decreased (hypersensitivity) (*P* = 0.000), and allodynia was found in some patients (*P* = 0.0001) (Table 3).

**Table 3:** Quantitative sensory testing parameters for the whole group.

	CDT °C (n = 95)	WDT °C (n = 93)	CPT °C (n = 93)	HPT °C (n = 93)	MDT mN (n = 93)	MPT mN (n = 96)	DMA NRS (n = 98)	PPT kPa (n = 96)
Test site mean(raw) SD	24.5 6.4	37.9 3.4	14.9 8.6	43.9 4.1	10.8 43.1	82.4 163.8	8.5 21.2	263.8 121.2
Control site mean (raw) SD	28.4 1.8	36.6 1.9	16.1 8.5	43.1 3.6	3.4 6.2	52.7 45.9	0 0	352.3 51.2
Mean intrapersonal difference SD	−3.97 6.07	−1.34 2.89	−1.19 7.82	−0.93 3.49	−7.47 43.2	−29.75 154.9	8.49 21.3	88.5 111.7
Difference direction	Negative	Negative	Negative	Negative	Negative	Negative	Present	Positive
Paired *t* test (intrapersonal difference)	<0.001	<0.001	0.1481	0.0369	0.0941	0.0630	<0.001	<0.001

CDT, cold detection threshold; CPT, cold pain threshold; DMA, dynamic mechanical allodynia; HPT, heat pain threshold; MDT, mechanical detection threshold; MPT, mechanical pain threshold; NRS, numerical rating scale; PPT, pressure pain threshold; WDT, warm detection threshold.

**Figure 4.**
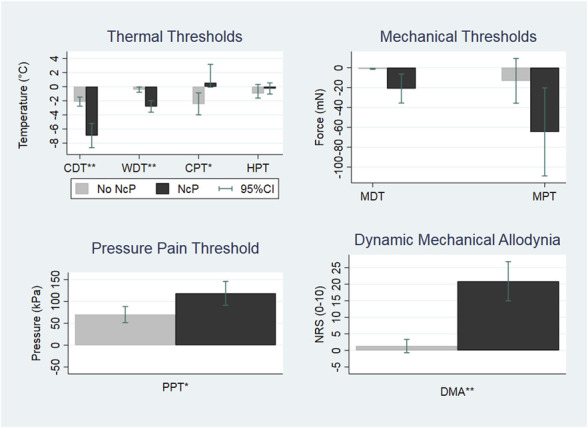
Mean within-subject differences for all QST variables (pain vs control site) of the patients classified as having neuropathic cancer pain (black) and not (gray) *(Positive values indicate gain of function, whereas negative ones indicate loss of function).* Data are presented as raw mean value of within-subject differences. Negative values indicate loss, whereas positive values indicate gain of function. The graph shows a predominant loss of sensory function (hypoesthesia) in terms of CDT and WDT and gain of function for CPT. Gain of function in the form of hyperalgesia to blunt pressure (PPT) is present and DMA (*P < 0.05, **P < 0.005). CDT, cold detection threshold; CPT, cold pain threshold; DMA, dynamic mechanical allodynia; HPT, heat pain threshold; MDT, mechanical detection threshold; MPT, mechanical pain threshold; PPT, pressure pain threshold; QAT, quantitative sensory testing; WDT, warm detection threshold.

Comparing patients with and without NcP, significant differences were found for those with NcP, characterized by hyposensitivity to cold and warm with a reduction of CDT and WDT, hypersensitivity to pressure, and hyperalgesia to cold with a reduction of PPT and CPT; the presence of DMA which was found only in patients with NcP (14 of 34) as seen in Figure 4. Mechanical pain threshold, mechanical detection threshold, and heat pain threshold did not show statistically significant differences. Both the unequal variance independent-samples *t* tests and Kruskal–Wallis test for the WSD of each QST variable in the group of patients with and without NcP revealed significant differences for CDT (*P* = 0.0032), WDT (*P* = 0.0018), CPT(*P* = 0.02), PPT (*P* = 0.02), and DMA (0.0001).

### 3.2. Characteristics of classifications' disagreement cases

Only 1 patient had a positive EAPC/IASP algorithm diagnosis with a negative gold standard classification, whereas 6 patients were negative at the EAPC/IASP algorithm evaluation and had a positive gold standard (Table 4).

**Table 4:** Characteristics of disagreements between the two methods used for neuropathic cancer pain assessment.

	EAPC/IASP	NeuPSIG criteria	Distribution		History		Diagnostic test		Simple bedside	Standardized bedside	Sensory alteration (QST)	S-LANSS score	NP adjuvant drugs
			Step A	Criterion 1	Step C	Criterion 2	Step C	Criterion 4	Step B	Criterion 3			
1	Yes NP	No NP	Y	Y	**Y**	**N**	N	N	Y	Y	Mechanical hypersensitivity	0	N
2	No NP	Yes NP	Y	Y	Y	Y	Y	Y	**N**	**Y**	Thermal hyposensitivity and mechanical hypersensitivity	10	Y
3	No NP	Yes NP	Y	Y	Y	Y	Y	Y	N	N	Thermal hyposensitivity	5	N
4	No NP	Yes NP	Y	Y	Y	Y	Y	Y	**N**	**Y**	Thermal hyposensitivity and mechanical hypersensitivity	6	Y
5	No NP	Yes NP	Y	Y	Y	Y	Y	Y	**N**	**Y**	Mechanical hypersensitivity	1	Y
6	No NP	Yes NP	Y	Y	Y	Y	Y	Y	**N**	**Y**	Mechanical hypersensitivity	19	N
7	No NP	Yes NP	Y	Y	Y	Y	Y	Y	**N**	**Y**	Hyposensitivity to thermal stimuli	6	Y

Disagreements in bold.

NeuPSIG, Neuropathic Special Interest Group; NP, neuropathic pain; QST, quantitative sensory testing; S-LANSS, self-reported Leeds Assessment of Neuropathic Signs and Symptoms.

The positive EAPC/IASP algorithm with a negative gold standard was due to the different evaluation made by the treating physician and the investigator; the first considered the history of the patient suggestive for the presence an etiological lesion, whereas the second did not. This patient had an S-LANSS score of 0. Six patients had a negative EAPC/IASP algorithm and positive gold standard assessment. In 5 patients, this was due to the results of the objective examination step: In 2 of them, thermal perception alone, which was assessed only within the gold standard, was abnormal; in 2 cases, mechanical perception alone was abnormal, whereas in 2 other cases, it was abnormal together with thermal perception. In the last case, the disagreement was due to the lack of objective sensory signs, which are required by the EAPC/IASP algorithm, whereas the NeuPSIG criteria allow for a probable diagnosis of NcP even without sensory signs.

Only one of these 6 patients had a positive S-LANSS score, whereas for the remaining 5 patients, the mean S-LANSS score was 5.5. Four of the 6 patients were receiving adjuvant drugs for NcP.

## 4. Discussion

In this study, we show that the EAPC/IASP algorithm^7^ has good sensitivity and specificity compared with the NeuPSIG criteria. The frequency of neuropathic pain obtained by using the 2 diagnostic algorithms was comparable 35.7% (95% CI 26.1%−45.3%) and 40.8% (95% CI 30.9%−50.7%) according to, respectively, the EAPC/IASP and NeuPSIG criteria. This finding compares with the upper limits of the prevalence previously reported.^3^ Although the patients were enrolled consecutively, a bias of selection might have occurred as also reflected by the rather high level of pain reported (average NRS in the past 24 hours and past 7 days = 6.2). NcP prevalence obtained by the S-LANSS was lower (18.4% [95% CI 10.6–26.2]), confirming previous evidence on the underestimation of NcP when using screening questionnaires.^11,25,32,36^

We found satisfactory sensitivity (85% [95% CI 70.2–94.3]) and specificity (98.3% [95% CI 90.8–100]) for the EAPC/IASP algorithm. The relatively lower sensitivity is explained by 6 false-positive cases (6.1%), which are mainly due to the disagreement on the identification of objective sensory findings (Table 4). In fact, in 4 cases, thermal hyposensitivity to cold stimuli was detected by applying the NeuPSIG algorithm, which, in our protocol, included the evaluation of thermal sensation, whereas it was not tested when applying the EAPC/IASP algorithm. The only case of false positives depended on a true disagreement in the interpretation by the 2 independent investigators of patients' history and, therefore, pain etiology. From the above results, we can say that the relatively lower sensitivity of the EAPC/IASP algorithm is likely to depend on the more accurate neurological examination used in applying the NeuPSIG criteria in this study.

The QST profiling showed sensory impairments in the painful sites in the overall group, characterized by hyposensitivity in the detection of thermal stimuli, both for cold and warm (*P* < 0.001). Hyperalgesia to hot pain stimuli (*P* = 0.0369), pressure pain stimuli (*P* = 0.000), and presence of allodynia (*P* < 0.001) were also encountered (Table 3). In the comparison of the group of patients with and without NcP, it was evident that these findings were mainly found in patients with NcP (Fig. 4) who had pronounced symptoms of deafferentation with sensory loss, especially to thermal stimuli.^4,26^ They also presented with allodynia and increased pain response to deep pressure stimuli, altogether suggestive of a more frequent impairment of a-delta and c fibers in the group of patients with NcP, consistent with the concept of a clinically relevant small-fiber neuropathy in NcP.

Despite limitations, QST's role in identifying sensory alterations and possibly stratifying patients for research purposes has been recognized,^2,28,37^ a recent review confirmed the lack of QST profiling data in patients with pain because of cancer, with studies mainly focusing on chemotherapy-induced peripheral neuropathies^24^ or on specific pain types such as bone and pleural pain.^23,24,31^ In these studies, sensory abnormalities to either thermal or mechanical stimuli were found in most patients with bone or pleural pain. Interestingly, 2 studies found alterations in the somatosensory profile of oncological patients who were not receiving chemotherapy or had no tumor-related neurological disturbances and/or pain.^21,22^ In these cases, paraneoplastic immune-mediated changes of the somatosensory system function might have been present.

This study describes 4 different levels of sensory profile assessment in patients with cancer pain: the S-LANSS questionnaire, the simplified neurological assessment made by the treating physician to apply the EAPC/IASP algorithm, the more comprehensive bedside neurological examination, and the QST, emphasizing the role of physical examination to provide an accurate diagnosis.^10,15–17^

The QST profiling of cancer pain showed an important impairment of thermal detection thresholds; yet in some cases, this will be difficult to notice in clinical practice. We believe that the use of a simple objective examination as in the EAPC/IASP algorithm will lead to the detection of most patients with sensory disturbances, as demonstrated also here, and it is easy to perform and not time-consuming. Our experience suggests also that for cases where a simple bedside assessment does not allow for a final decision, thermal perception should be assessed before ruling out sensory abnormalities.

To the best of our knowledge, this is the first time that criteria adapted for patients with cancer pain have been applied to diagnose NcP. It is also the first report on an QST assessment in patients with cancer pain with or without a neuropathic component. Patients on the same day of the enrollment and investigators were blinded to the results of the other's evaluation to reduce bias. Steps were provided to guide and optimize the use of the EAPC/IASP algorithm in everyday clinical practice.

Some limitations need to be acknowledged. This was a cross-sectional study performed in a tertiary national referral center including only patients with pain because of cancer, avoiding the inclusion of those with documented non–cancer-related neurological abnormalities and conditions such as chemotherapy-induced peripheral neuropathies. These, and other exclusion criteria, can affect on the generalizability of the results. Neuropathic cancer pain prevalence data reported here are relevant to the target population under study, may have been influenced by potential selection bias, and should therefore viewed with caution, also considering that prevalence estimate was not an objective of the study. The analysis of the QST data, because of the lack of normative values for some of the testing sites and the special characteristics of the oncological population, was analyzed using the within-subject differences. In addition, unlike neuropathic pain from other causes, NcP is rarely due to a selective lesion of nervous tissues alone, with the tumor usually affecting one or more additional tissues^19,26,33^ (Table 2), possibly leading to mixed pain mechanisms (nociceptive and neuropathic). A number of patients (37%) were receiving chemotherapeutics, and although they had no diagnosis or suspected CIPN, this needs to be acknowledged as a possible limitation and confounding factor for the QST results. Because some sensory alterations were also noted in the control sites, it would have been relevant to have a control group. In addition, considering most patients were receiving analgesics and adjuvant drugs, it could be that some patients' responses and sensory findings could be modified by their effect. Yet, in these populations, it would be very difficult to study patients without any analgesic treatments.

## 5. Conclusions

Previous evidence has demonstrated the need for an appropriate assessment of cancer pain,^4,9,33^ especially NcP, which is often related to worse analgesic outcomes and need for specific adjuvant drugs.^12^ This study highlights the role of choosing adequate criteria for NcP diagnosis and their application in clinical practice. The lack of standardized approaches for its evaluation can affect treatment decisions in clinical practice and limit results on drugs benefit in research and clinical trials. Reproducibility of the results on the EAPC/IASP algorithm could help confirm generalizability making this diagnostic tool, together with more mechanistic insights on pathophysiology, instrumental for future clinical and research advancement in cancer pain.

## Disclosures

E.Z. has received honoraria from Amgen. R.R. has received fees as a speaker or for counseling services from the following companies: Aristo Pharma, Cannamedical, Hormosan, Grünenthal, Lilly & Company, Pfizer, Tilray, and Spectrum Therapeutics. S.K. has received honoraria from Nutricia Norge AS and Pfizer Norge AS. A.C. has received honoraria from Molteni, Pfizer/Eli Lilly Italia Spa, and Mundipharma. All other authors have no conflicts of interest to declare.

## Appendix A. Supplemental digital content

Supplemental digital content associated with this article can be found online at http://links.lww.com/PR9/A221.
